# Duties of Candour in Healthcare: The Truth, the Whole Truth, and Nothing but the Truth?

**DOI:** 10.1093/medlaw/fwac004

**Published:** 2022-03-21

**Authors:** Oliver Quick

**Affiliations:** Centre for Health, Law and Society, University of Bristol, Bristol, UK

**Keywords:** Apologies, Candour, Harm, Law, Regulation, Safety

## Abstract

The creation of professional and statutory duties of candour has formalised the requirement for clinicians and healthcare organisations to be honest with patients and families when treatment has gone wrong. This article explains the background to creating both duties, analyses the concept of candour, the role of apologies, and considers evidence about compliance. It argues that making candour a statutory requirement appropriately reflects the ethical imperative of telling the truth about harm and is a powerful signal for honesty. However, being candid is not easy in the context of complex professional cultures, the realities of delivering care in under-funded health systems, and in the shadow of possible legal and regulatory proceedings. Proposals in the current Health and Care Bill to create investigatory ‘safe spaces’ which prohibit the disclosure of information submitted to the Health Service Safety Investigations Body undermine candour. This article argues against such proposals, which are both wrong in principle and highly problematic in practice. Candour should be respected as a cardinal principle governing not only the conduct of those providing care, but also those who investigate such incidents. Harmed patients and their families deserve to know the whole truth.

## I. INTRODUCTION

Healthcare harm is a global public health problem. The World Health Organization estimates that adverse events cause more deaths than lung cancer, diabetes, or road injuries, and that 80% of adverse events are avoidable.^1^ In low- and middle-income countries, poor-quality healthcare accounts for 10–15% of deaths annually.^2^ Such statistics are striking if slightly simplistic in that unsafe care combines with pre-existing health conditions and diseases, and avoidability assessments are likely based on ideal, rather than real-world conditions. Nevertheless, in England alone, the additional annual financial cost of providing further care to harmed patients would equate to employing over 2,000 salaried general practitioners (GPs) and 3,500 hospital nurses,^3^ much needed given the high number of vacant positions in the National Health Service (NHS) workforce.^4^ The annual cost of compensating and managing maternity negligence cases (£2.1 billion) now exceeds the amount spent on delivering babies (£1.9 billion).^5^ With £83.4 billion ‘set aside’ for settling future liabilities, managing medical negligence is one of the most substantial public sector financial liabilities faced by the UK government.^6^ Remarkably, there remains no coherent cross-government strategy and policy to address these spiralling costs.^7^

Behind the statistics are patients, their families, and clinicians who all suffer, sometimes in silence, after being affected by such harm. For patients and their families, a lack of openness in the aftermath of an adverse event adds emotional insult to their physical injuries, often having a long lasting impact.^8^ Despite the clear importance of honesty in healthcare, classic codes of medical ethics such as the Hippocratic Oath and the Declaration of Geneva are strangely silent on truthfulness.^9^ Whilst medical ethics textbooks contain entries on honesty, veracity, truth-telling, openness, and transparency,^10^ discussion has surrounded the difficulties of communicating devastating diagnoses to patients, rather than disclosing medical harm. Historically, being open to patients about error has never been normalised in medicine.^11^

However, the creation of professional^12^ and statutory^13^ duties of candour has formalised the requirement for clinicians and healthcare organisations to be honest with patients and families when treatment has gone wrong. Being honest about healthcare harm has important implications for patients, families, clinicians, and the health service as a whole. This applies both to raising concerns about colleagues or the safety of services, often referred to as whistle-blowing, and to clinicians openly disclosing and adverse events to their patients. Both raise sensitive issues of professional identity, reputation, loyalty, and trust. Whilst there may be overlap between situations calling for staff to speak up and to say sorry themselves, this article focuses on the latter.^14^ It explains the background to creating both duties and analyses the detailed terms of candour (Section III) before considering evidence about compliance (Section IV). It argues that making candour a statutory requirement appropriately reflects the ethical imperative of telling the truth about harm and is a powerful signal for honesty. However, the call for candour arises in the context of complex professional cultures and the realities of delivering care in under-funded health systems and a busy regulatory and medico-legal landscape.

Being candid is not easy, but, as discussed in the Section II, it is definitely the right thing to do. Candour should be respected as a cardinal principle governing not only the conduct of those providing care, but also those who investigate such incidents. Proposals to create investigatory ‘safe spaces’ that prohibit the disclosure of protected information, contained in the current Health and Care Bill,^15^ undermine candour by withholding information from patients and families. Whilst compromising candour might be considered an acceptable trade-off in order to ensure greater learning and improvement following safety incidents, this article argues that such reforms are both wrong in principle and highly problematic in practice.

## II. THE VALUE OF HONESTY IN HEALTHCARE

The duty of healthcare professionals to be honest with patients is a fundamental part of ethical treatment. Openness and honesty are central to trust in clinicians and health systems.^16^ Patients value professional openness and honesty in relation to discussing diagnosis, prognosis, and the risks and benefits of different treatment options available to them, which, of course, is a legal requirement.^17^ Openness is also prized beyond the private patient–clinician relationship in relation to key public health issues; for example, the safety of medicines and medical devices,^18^ the funding (or not) of treatments,^19^ the procurement of NHS goods and services,^20^ clinical trials data,^21^ and decision making about vaccine prioritisation.^22^ Transparency is also important in terms of clinicians disclosing the truth to patients and their families, and to processes for investigating incidents and complaints.

Honesty is a celebrated character trait of virtuous (and courageous) professionals and is central to therapeutic relationships.^23^ Telling patients the truth about harm is consistent with both consequentialist and duty-based approaches to medical ethics. There is a clear utility in being honest with patients and their families in terms of understanding what went wrong and why. The desire for truthful explanation and, where appropriate, apology and accountability are entirely understandable expectations in the aftermath of harm. Research has long suggested that these desires motivate the majority of complaints about healthcare treatment.^24^ This has been validated by a recent survey which found that explanation, apology, and prevention were the dominant reasons for clinical negligence claims, with prevention cited as the most prominent *primary* reason.^25^ The idea that openness about harm may also be beneficial to healthcare professionals has received much less attention. Despite powerful examples of clinicians sharing their suffering in the aftermath of medical error,^26^ and revealing the emotional toll of inadvertently harming patients, such openness has generally been regarded as a high-risk low-reward activity. Furthermore, clinicians have generally explored the emotional aspects of medical harm, including self-forgiveness, in protected professional spaces where patients are not present.^27^

In terms of the ‘four principles’ of biomedical ethics,^28^ telling patients the truth respects their autonomy to understand what has happened to them in the course of healthcare treatment. This article takes the position that nobody has a stronger claim on the truth than those who are the primary victims of harm. This is neatly encapsulated in the oft-quoted mantra ‘nothing about me without me’.^29^ Autonomy can also be extended to respecting patient choice in responding to harm; for example, whether to forgive, complain, or even litigate. Non-maleficence, drawing on the first principle of medical practice and ethics (first of all, do no harm), recognises the emotional damage to patients who are denied the truth. Beneficence requires a positive duty for doing good. In this context this means communicating compassionately and candidly with patients. In short, candour should be a fundamental part of duties of care in therapeutic relationships. The concept of justice introduces more scope for conflict, given that honesty has implications for patients, professionals, and also health systems, and that different positions on what represents a just outcome are inevitable. Justice has generally been explored in terms of fair and equal allocation of healthcare resources, and largely examined in terms of who gets treatment and the legitimacy of allocative decision making.^30^ This article argues that there should be no scarcity of the resource in question here—the truth—to which patients and families should have full and fair access.

In terms of duty-based ethical approaches, it is tempting to regard honesty as an absolute principle for healthcare professionals. If not, then what trumps truth telling? For some, an absolutist position risks overlooking the ‘nuances and necessities’ of clinical practice, which may warrant withholding the truth for good reasons, mainly where patients prefer not to know or where knowing might be more harmful.^31^ However, the case for a stringent moral position is arguably stronger when applied to harm caused by unsafe care. The appropriateness of ‘therapeutic privilege’ assessments of disclosure possibly doing more harm than good, is doubtful in the context of disclosure of risks *before* consenting to treatment,^32^ and is surely less justifiable when treatment has gone wrong. This article argues that a strong presumption in favour of honesty with patients should apply, and any attempts to dilute the commitment to candour should be subject to careful scrutiny and only considered in rare and narrowly conceived therapeutic privilege situations.^33^ Whilst medical knowledge and, indeed, the delivery of healthcare is ‘engulfed and infiltrated by uncertainty’,^34^ and circumstances and context can be complex, there is no justification for being economical with the truth about healthcare harm to patients and their families.

On a broader population health level, openness permits greater opportunities for studying, learning, and preventing healthcare harm. This has been a dominant theme running through the patient safety literature, policy development, and inquiry reports for over two decades.^35^ However, whether honesty is a financially costly policy, in terms of managing increased complaints and claims, remains an open question. The relationship between candour, litigation risk, and financial cost is poorly understood. Studies from the USA suggest that it would be wrong to assume that greater openness necessarily leads to increased claims and costs. Indeed, evidence from ‘communication and resolution’ programmes in the USA, demonstrate that honesty does not lead to higher liability costs, and may even reduce costs where open disclosure is accompanied by proactive compensation.^36^

Encouraging openness has been a prominent feature of policy initiatives and guidance issued by the UK’s Department of Health and Social Care. In England, the now defunct National Patient Safety Agency issued a best practice framework about ‘Being Open’ in 2009.^37^ NHS Resolution (NHSR) has also advised staff to say sorry and reminded them that apologies are not admissions of liability.^38^ The value of openness also features prominently in the NHS Constitution,^39^ with numerous references to expectations of transparency and patient and family involvement in care and incident investigations. In principle, the commitment to openness appears comprehensive, yet in practice there remains a considerable ‘disclosure gap’. This mismatch between patient expectations and professional practice has been explained by reference to four main areas: (i) acknowledging harm is psychologically difficult and conflicts with a professional identity as healers; (ii) a lack of training on how to communicate with compassion and candour; (iii) underestimating how important full disclosure is to patients; and (iv) fear of litigation and a poor understanding of law and legal process.^40^ Conversely, the following five themes appear to encourage honesty: (i) open disclosure as a moral and professional duty; (ii) positive past experiences; (iii) understanding the repercussions; (iv) role models and guidance; and (v) clarity.^41^ The next section will examine how candour has been incorporated in professional and statutory duties.

## III. DUTIES OF CANDOUR: PROFESSIONAL AND STATUTORY

The concept of candour has escaped significant analysis in ethical and legal literatures. Ethical discussion has tended to refer to candour alongside openness, honesty, and transparency, and seldom distinguished between them.^42^ Arguably, candour is more than just a synonym for truth or honesty and is distinct in requiring *complete* openness and frankness. Crucially, in this article, candour is conceived as requiring full frankness about not only the nature of healthcare harm, but also the explanations for it, which may require further investigation and take time to understand. This article argues that the concept of candour imposes both a private and a public duty on clinicians and the health service. The *private duty* for clinicians to be open and honest with their patients is now clearly prescribed in regulatory guidance examined below and is predicated on respecting the autonomy and dignity of patients to know *how* they have suffered harm. More significantly, the statutory duty of candour also imposes a *public duty* on the health service to fully respect openness and transparency when delivering treatment and services. This is arguably analogous to principles of procedural justice in administrative law,^43^ especially in terms of the obligation for giving reasons, in this case explaining *why* harm happened. It is appropriate to conceive of candour as imposing a public law obligation as this serves the public interest in two ways: it should facilitate learning from failures and also enhance public confidence in healthcare by requiring openness and honesty.

Candour has occasionally, and fleetingly, featured in judgments in clinical negligence cases. For example, in *Lee v South West Thames Regional Health Authority*, Sir John Donaldson MR stated that ‘some thought should be given to what is the duty of disclosure owed by a doctor and a hospital to a patient after treatment,’^44^ albeit that this issue was not central to the appeal in that case. Two years later, in *Naylor v Preston*, the same judge went further in stating that ‘in professional negligence cases, and in particular in medical negligence cases, there is a duty of candour resting on the professional man’.^45^ However, the tort of negligence has not evolved to oblige openness after, as well as before, treatment;^46^ nor has the common law developed a free-standing duty of candour. Whilst candour has long been a central recommendation of key public inquiries and policy reviews,^47^ it has only recently evolved into an ethical and legal concept expressed in regulatory and statutory form.

Openness and honesty are now obliged by health professional regulatory codes of practice (the professional duty of candour) and also in legal form (the statutory duty of candour). Whilst the general thrust of both duties is the same, they differ in scope, application, and enforcement. The *professional duty* applies to clinicians and includes low harm or even near misses. The *statutory duty* applies to ‘health service bodies’, which primarily means NHS trusts and organisations regulated by the Care Quality Commission (CQC). Placing the statutory duty on organisations rather than individuals is appropriate in terms of encouraging policies and procedures and avoids placing undue pressure on clinicians. These bodies are required to be open and transparent with patients or their representatives about their care, and to notify, support, and provide a truthful account, to advise and apologise in relation to ‘notifiable safety incidents’.^48^ Neither duty makes provision for therapeutic privilege decisions not to disclose, beyond noting that some patients may request not to know the details of the incident.^49^ The background to creating both duties and the detailed terms of candour will now be considered, before examining evidence about compliance in Section IV and proposals which seem to contain candour in Section V.

### A. Professional Duty of Candour

The professional duty of candour was created as a response to the death of Robbie Powell, aged 10, in 1990 of undiagnosed Addison’s disease, a rare auto-immune disorder of the adrenal glands. Although a paediatrician suspected Addison’s disease as a possibility, and wrote to Robbie’s GP advising on testing and referral, neither happened and there were many missed opportunities to prevent his deterioration and death. None of the clinicians informed the family that Addison’s was suspected. Will Powell, Robbie’s father, sought to uncover the truth surrounding his son’s preventable death. There was evidence that two GPs had forged a referral letter to make it appear that it was written before Robbie died, and also amended his medical notes giving the misleading impression that they were written contemporaneously.^50^ The Health Authority admitted liability in negligence and paid damages of £80,000 to the family. There followed an unsuccessful action for psychiatric injury, which failed for lack of proximity.^51^ The European Court of Human Rights also rejected an argument under Article 2 of the European Convention on Human Rights, in relation to the need for an effective investigation into the circumstances leading to Robbie’s death, largely based on the dubious grounds that settling the clinical negligence claim constituted an adequate investigation. The Court’s judgment that

as the law stands now … doctors have no duty to give the parents of a child who died as a result of their negligence a truthful account of the circumstances of the death^52^

made for difficult reading.

Nevertheless, Will Powell campaigned for ‘Robbie’s Law’, a legal obligation for all healthcare providers to be truthful with patients and families after an adverse event.^53^ The General Medical Council (GMC) responded by amending its code of conduct, in 1998, stating that if a patient has suffered serious harm, professionals ‘should act immediately to put matters right … explain fully to the patient what has happened … [and] when appropriate you should offer an apology.’^54^ With direct reference to Powell’s case, the GMC said that ‘if a patient under 16 has died you must explain … the reasons for, and the circumstances of, the death to those with parental responsibility.’^55^ These provisions were modified in the GMC’s guidance applicable from 2001 to 2006, with a separate section on ‘Being open and honest with patients if things go wrong’ introduced. This extended the obligation to situations where the patient had suffered harm or distress, and stated that an apology and explanation should be offered.^56^

In 2015, a more detailed and demanding set of obligations were contained in jointly written guidance by the GMC and the Nursing and Midwifery Council.^57^ This concerns the professional duty of candour which applies to individual registrants and notes that patients have a ‘right to receive an apology from the most appropriate team member regardless of who or what may be responsible for what has happened’.^58^ This is significant in terms of requiring apologies for matters potentially beyond a professional’s control and responsibility. The guidance also recommends that apologies are recorded on clinical notes and are followed up in writing. The call for candour is extensive—applying when something has gone wrong with care, including the materialisation of known complications, and also suggesting that near misses might be disclosed, albeit leaving that to professional discretion.^59^ Whilst this guidance was a welcome commitment to the importance of candour by professional regulators, it was soon followed by the more important and higher profile statutory duty of candour.

### B. Statutory Duty of Candour

Regulation 20 of The Health and Social Care Act 2008 (Regulated Activities) Regulations 2014 places candour on a statutory footing. This was a response to recommendation 181 of the Francis Report into the events at the Mid Staffordshire NHS Trust, which called for a statutory duty of candour on healthcare providers and registered healthcare professionals who believe or suspect that treatment or care has caused death or serious injury.^60^ As examined below, the enacted duty is different in two important respects: it only applies to organisations but it extends beyond death and serious injury to include moderate harm. Similar versions have followed in Scotland^61^ and Wales,^62^ and proposals in Northern Ireland and Ireland are also in progress, following high-profile inquiries into the deaths of five children after receiving intravenous fluids^63^ and failures in a cervical cancer screening programme, respectively.^64^ This article focuses on the English statutory duty of candour, although the impact of the different national duties of candour will be worth monitoring.

As with much modern-day statutory drafting, Regulation 20 is a lengthy provision made up of nine clauses. ‘Honesty’ has been translated into legal complexity. The duty came into force in November 2014 and initially applied only to ‘health service bodies’, which means health and social care organisations registered with the CQC. Primary care organisations, dentists, private healthcare, and adult social services were initially excluded and subsequently brought within Regulation 20 from April 2015, albeit with a different harm threshold for triggering the duty. There are two parts to the statutory duty. First, Regulation 20(1) imposes a general requirement for ‘registered persons’ to be open and transparent with patients or their representatives about care and treatment. This reflects the aim of creating a culture of candour which has long been identified as crucial to improving patient safety. In the words of the influential Williams and Dalton report, a ‘culture of candour is a culture of safety, and vice-versa’.^65^ Secondly, there are specific reporting requirements placed on providers in relation to ‘notifiable safety incidents’, defined as any ‘unintended or unexpected incidents’ that could result or appear to have resulted in death, severe, moderate, or prolonged psychological harm.^66^ The coverage of the duty is broad in extending beyond ‘mistakes’ or ‘failures of care’ to include harm arising from known risks communicated as part of the informed consent process. The specific requirements involve notifying, supporting, providing a truthful account, advising, and apologising to patients and/or families who have suffered the requisite harm as a result of such an incident.^67^ This must be done as soon as is reasonably practicable after becoming aware of the incident, and such communication must be followed by written notification.^68^

It is these detailed terms of candour that are of particular interest to clinicians, managers, regulators, and researchers. Setting the appropriate harm threshold for making an incident notifiable was the subject of pre-legislative Department of Health (DH) commissioned review.^69^ Many who opposed the duty argued in favour of confining it to cases involving death or severe harm. The argument that honesty should depend on the degree of harm suffered by patients was unprincipled and ultimately rejected in the DH review. For hospital care, a ‘notifiable incident’ is ‘any unintended or unexpected incident that could result in, or appears to have resulted in death, severe, moderate or prolonged psychological harm.’^70^ In fact, the term ‘moderate’ is itself slightly misleading, in that it includes significant harm such as:

unplanned return to surgery, an unplanned readmission, a prolonged episode of care, extra time in hospital or as an outpatient, cancelling of treatment, or transfer to another treatment area (such as intensive care).^71^

For primary care organisations, dentists, private healthcare, and adult social services there is no requirement for them to inform patients about incidents that ‘could’ result in significant harm but have not yet done so. According to the CQC, the:

definitions have been differentiated in this way to account for the different notification systems for health service bodies and all other providers. In doing so, they are intended to reduce the administrative burden caused by the introduction of this new statutory duty of candour.^72^

Regrettably, this distinction effectively permits a weaker form of candour outside of NHS secondary healthcare. The duty does not apply to harm deemed to fall below moderate, or to near misses. In both settings, the guidance envisages what we might call ‘long candour’, in that it continues to apply when new information emerges, *regardless* of when the incident occurred and irrespective of the litigation process.^73^ Nevertheless, as explored below, the relationship between candour and the medico-legal landscape remains somewhat uncertain.

It is striking that the statutory duty mandates an apology which is described as an ‘expression of sorrow or regret’.^74^ This description differs from the accepted definition of a ‘full apology’ in making no reference to the acknowledgment of causing harm or offence.^75^ As van Dijck has neatly summarised, there are three components of full apologies: affect (regret, remorse), affirmation (admission of fault), and action (compensation, reparation).^76^ Expressing sorrow or regret, whilst an important part of the healing process, is at best a partial apology.^77^ Apologies that are only given in order to comply with a court order or a statutory duty may be described as ‘ordered’ apologies.^78^ This is not necessarily inappropriate in that full apologies may not be needed for every incident triggering the statutory duty of candour. The best example would be the materialisation of potential complications which patients were warned about during the informed consent process and which were not caused by any clinical failings. Whilst such an example merits full candour about the nature and causes of harm, it is difficult to see why it would require a full apology. Arguably, even harm that is associated with sub-standard care should not automatically warrant a full apology. Given that medical harm is largely a product of poor conditions—understaffed, poorly resourced risky settings—clinicians may feel that they bear no personal responsibility and hence have nothing to apologise about. Indeed, some may understandably resent appearing to take responsibility for unsafe systems that are the root cause of much medical harm and beyond their control.

Apologies are complex, unique interactions that depend on the needs of those involved. As Lazare describes so well, apologies:

have the power to heal humiliations and grudges, remove the desire for vengeance, and generate forgiveness on the part of the offended parties. For the offender, they can diminish the fear of retaliation and relieve guilt and shame … the result of the apology process, ideally, is the reconciliation and restoration of broken relationships.^79^

Sincere apologies can strengthen the moral community within which they are made by validating what the community regards as morally wrong.^80^ However, an effective apology process is complex as it requires time for active listening, understanding, and sensitive communication. Genuine apologies require an ethical commitment as well as emotional intelligence and effective staff training and support. In reality, many apologies are partial, half-hearted, and fail to hit the spot. Recent behavioural insight research examining the motivation of clinical negligence claimants found that only 31% of respondents felt they received an apology and only a minority regarded it as a proper apology.^81^

Apologies raise important questions for law and legal systems which have yet to be fully understood.^82^ In England and Wales, despite section 2 of the Compensation Act 2006 re-stating that apologies are not (on their own) admissions of liability or breaches of statutory duties, there is no evidence that such provisions have improved the rate and quality of apologies. Indeed, very little is known about the impact of this statutory provision, and it is regrettable that the Ministry of Justice has not evaluated its impact. The stated reason for this is that it would involve examining the ‘basis on which the courts have reached their decisions in a wide range of individual cases’, and that this might ‘undermine the independence of the judiciary and cast doubt on the way in which they have interpreted the law’. ^83^ It is hard to see why such research would necessarily have such implications, and this response also overlooks the fact that the vast majority of such disputes are settled out of court. As Leung and Porter have noted, section 2 of the 2006 Act lacks clarity and comprehensive coverage which may hamper effective implementation of the statutory duty of candour.^84^ More generally, there are no clear conclusions on the liability impact of apology laws around the world, although there is increasing evidence that apologies which form part of broader and timely redress packages do not lead to increased financial costs.^85^

## IV. CANDOUR COMPLIANCE

Professional regulators have long struggled to effect behavioural change, and encouraging candour is no exception.^86^ A lack of candour about healthcare harm does not feature directly in the determinations of fitness to practise panels, which have, instead, dealt with cases involving failing to disclose convictions or financial dishonesty.^87^ The need for greater consistency and clarity about standards of candour was identified when the professional duty was created.^88^ An evaluation of the progress of professional regulators in embedding the duty in practice demonstrates the difficulties of normalising candour. Measuring candour quantitatively remains challenging, and research to date has focused on analysing questionnaire and focus group responses of regulators and key stakeholders. A recent review by the Professional Standards Authority for Health and Social Care (PSA) has identified five main barriers to enhancing candour: (i) toxic work environments of blame and defensiveness; (ii) lack of time to be candid with patients (especially given staff shortages); (iii) education and training (about communication skills and to display myths about the legal implications of apologising); (iv) fear of complaints and litigation; and (v) communication.^89^ That these barriers have remained static suggests that professional regulation has had limited, if any, impact on instilling a culture of candour and validates the decision to create the statutory duty which has captured more attention.

Placing candour on a statutory footing encountered considerable resistance by medical defence unions, who claimed that the professional guidance was sufficient.^90^ Obliging candour in law was also resisted from many engaged in patient safety research, based on a cautious view that it might be counterproductive by discouraging clinicians from being open and honest.^91^ Whilst the issue of accurate understanding and communication of legal duties is important, this remains an unduly pessimistic prediction of the impact of law. It discounts the potential normative contribution that legislation can make to the culture of healthcare provision. This negative perception about law reflects the prevailing view from the patient safety movement, which has tended to deny a positive role for law.^92^ More broadly, the role of law as a determinant of health and well-being has been under-recognised and researched.^93^ In terms of candour, unlike the soft law mechanisms of guidance and policies, a statutory duty has greater capacity for capturing attention and contributing to behavioural change. This positive prediction about the impact of the duty has been supported somewhat by anecdotal evidence of increased reporting to patients and staff reminding colleagues about the legal obligation.^94^ The requirement for NHS staff to have full knowledge about candour regulations as part of the patient safety syllabus should also ensure greater understanding of what the duties require.^95^ Nevertheless, it must be acknowledged that the relationship between legal duties and the safety of healthcare is complicated, relatively poorly understood, and requires robust empirical health law research.^96^ In particular, mixed methods research is needed to investigate the impact of the duties of candour, both on professional practice and patient experience, and to identify and promote good practice around candid communication.

The statutory duty is enforced by the CQC which may remove a provider’s registration, impose conditions, issue warnings, requirement notices and fines, and bring prosecutions. The CQC has no specific approach to monitoring compliance with the duty and approaches it as part of its inspection of whether good care is being provided.^97^ There remains weak evidence on compliance with the duty, and our understanding of its impact remains limited. The only evidence to date is based on reviews of CQC inspection reports from 2015 to 2017, which rely on comments by each Trust about their own implementation of the duty and, thus, lacks independence and rigour. In 2015, of the 90 reports analysed, only 13% made detailed reference to the duty, with the remaining making ‘moderate’ (61%), ‘superficial’ (19%), and no (7%) reference to the duty.^98^ Out of 34 examples where the reports criticised candour implementation, 20 of these had no accompanying recommendation to improve, and the CQC provided no information on how Trusts had responded to such recommendations. Encouragingly, a follow-up review in 2018 found a markedly higher percentage of reports with detailed analysis of candour (39%) which might suggest that the statutory duty is starting to have some impact in practice.^99^

Concerns about governing candour through compliance rather than professionalism were also expressed before the duty was created. The Berwick review into patient safety, commissioned as part of the response to the Francis Report, noted that ‘culture will trump rules, standards and control strategies every single time’.^100^ In a similar vein, the Williams and Dalton review predicted that a:

compliance-focused approach will fail. If organisations do not start from the simple recognition that candour is the right thing to do, systems and processes can only serve to structure a regulatory conversation about compliance.^101^

The challenge of adopting an appropriate style of regulation and finding a synergy between persuasion and punishment has been a key theme of regulatory theory and practice. The concept of ‘responsive regulation’ maintains that regulators must understand the context and culture of the field being regulated, and pursue soft supporting nudges rather than command and control measures.^102^ Early evidence about enforcing candour suggests that a light touch approach has been favoured, largely through issuing ‘requirement notices’ to provide adequate staff training about the duty.

In 2018, following a request from the charity Action against Medical Accidents, the CQC confirmed that it had taken 15 actions against NHS Trusts and 90 against primary care and private care providers, in relation to the statutory duty.^103^ The approach of the CQC, akin to the Health and Safety Executive, has largely been to prosecute as a last resort.^104^ However, Regulation 20 allows the CQC to proceed directly to criminal enforcement action without first issuing a warning, and a tougher approach to enforcing the duty appears now to be emerging.^105^ In January 2019, Bradford Teaching Hospital NHS Foundation Trust was fined £1,250 for failing to apologise to a bereaved family following the death of a baby within a ‘reasonable’ time.^106^ Royal Cornwall Hospitals was fined £16,250 for 13 breaches of the duty of candour in October 2019, after failing to notify patients or their family of the facts available as soon as reasonably possible.^107^ The first case to go to court resulted in University Hospitals Plymouth NHS Trust being fined £1,600 after failing to disclose details relating to a surgical procedure or apologise, following the death of a 91-year-old patient.^108^

It is regrettable that the English statutory duty contains no obligation for training and supporting staff on how to communicate candidly and cope with the emotional aspects of such work. The symbolic importance of making candour a legal obligation should have been accompanied by making effective training and support compulsory. The equivalent duty in Scotland obliges providers to provide training and support for staff who carry out the duty of candour procedure.^109^ There is an abundance of evidence about the emotional toll that adverse events have on clinician ‘second victims’.^110^ It is also clear that engaging and supporting clinicians is crucial to the success of new patient safety initiatives.^111^ Qualitative research into the experiences of clinical and managerial leaders about implementing open disclosure initiatives suggest that they may be a ‘hard sell’ to colleagues working in sub-optimal conditions. Cultural work ‘to explain the benefits of candour’ is essential in seeking to embed behavioural change, but extremely challenging given entrenched attitudes and assumptions about the consequences of being open. This is not just a culture of concealment, but also a ‘normalised incuriosity’ which is difficult to disrupt.^112^ Nevertheless, the power and profile of a statutory duty remains an important trigger for challenging the norm of non-disclosure.

## V. CONTAINING CANDOUR?

Being candid to patients and families is not easy, and confronts complex cultural issues around professional identity, reputation, and the fear of being unfairly blamed. The duties do not exist in isolation. They interact with a medico-legal landscape dominated by the clinical negligence system and other means of redress via complaints processes and, less commonly, criminal and coronial investigations.^113^ The relationship between candour and the risk and costs of litigation is particularly important with the overall cost of compensating and managing claims in England in 2020–21 amounting to £2.2 billion, with £600 million being spent on legal costs.^114^ Given that the National Audit Office has estimated that only 4% of those who suffer a harmful incident in healthcare make a claim,^115^ and that academic analysis has doubted that there is a ‘compensation culture’ in the UK,^116^ there remains real scope for a growth in the number and cost of claims. Uncertainty about the financial costs of candour and a desire to protect public resources has been the dominant concern driving policy proposals from the DH.^117^

The key organisation here is NHSR, which has the somewhat conflicted remit of reducing costs, compensating those harmed by clinical negligence, *and* seeking to support candour.^118^ It undertakes numerous functions, the most relevant of which is administering clinical indemnity schemes on behalf of the NHS. The main scheme is the Clinical Negligence Scheme for Trusts (CNST), a risk pooling scheme made up of 530 NHS Trust members which operates on a not-for-profit and pay-as-you-go basis with no limits or excesses. The scheme collects annual membership subscriptions from each Trust to cover the projected costs of the scheme in that year, which are calculated based on: (i) a risk-based element (staffing size and activity levels); (ii) claims experience over past 5 years; and (iii) known outstanding claims. Obstetric claims arising from birth injuries represent 59% of the total value of all claims,^119^ and have been the focus for attempts at prevention and learning.^120^ In particular, NHSR has sought to incentivise safer maternity care by allowing Trusts that demonstrate compliance with 10 safety actions to recover the element of their contribution to the CNST maternity incentive fund. For example, safety action 10 requires that Trusts report cases of severe brain injury to NHSR’s Early Notification Scheme and makes it clear that lack of compliance can lead to referral to the CQC.^121^

The Early Notification Scheme was established in 2017 to encourage Trusts to notify NHSR of maternity incidents of severe birth injury within 30 days of incidents. It is designed to allow NHSR to manage claims more efficiently by enabling earlier investigation and settlement of cases that are deemed eligible for compensation.^122^ The scheme, which is similar to the ‘communication and resolution’ programmes in the USA,^123^ aims to support the duty of candour by encouraging explanations and apologies. A review from the first year of the scheme found that only 77% of Trusts had notified families about such incidents, which are all highly likely to be ‘notifiable safety incidents’, even though they had notified NHSR within 30 days of the incident. Furthermore, only 30% of families had been invited to be involved in investigations.^124^ This reveals a disappointing level of compliance with the statutory duty of candour. The requirement for Early Notification Scheme was paused from 1 April 2020 due to the pressures of the COVID-19 pandemic, with Trusts instead asked to notify the Healthcare Safety Investigations Branch (HSIB) to conduct learning investigations and then refer cases back to NHSR to examine the legal implications.^125^

From the perspective of patients and society, there is a clear private and public interest in ensuring openness and honesty around healthcare harm. However, there are also concerns that clinicians may be reluctant to risk being open, for fear of disciplinary or legal consequences, which may limit the effectiveness of investigations and the capacity for learning. A difficult question arises over whether candour to patients should be compromised in order to protect staff from such risks and enable better quality investigations. Such concerns have led some jurisdictions to create qualified privilege laws to encourage ‘blame free’ reporting and to protect practitioners from legal repercussions.^126^ No such specialist qualified privilege laws exist in the UK, although considerable concern has been expressed about the need to allow a confidential space shielded from legal or disciplinary processes,^127^ and for achieving a ‘just culture’ which balances safety and accountability.^128^

These concerns have informed proposals to introduce qualified privilege by prohibiting the disclosure of material gathered or generated as part of safety investigations.^129^ The Health and Care Bill, currently before Parliament, contains clauses which address the poor quality of NHS safety investigations by placing the HSIB on a statutory footing as an independent agency,^130^ and changing its name to the Health Service Safety Investigations Body (HSSIB).^131^ The proposals are inspired by models used in other safety-critical industries, most notably aviation with national agencies such as the UK Air Accidents Investigation Branch. Such safety specific agencies draw on expert skills and knowledge and focus on learning and improvement rather than blame.^132^ They typically observe a duty *not* to disclose information obtained during investigations, in order to protect those who provide evidence.^133^ Drawing on this model, clause 108 of the Health and Care Bill states that ‘The HSSIB, or an individual connected with the HSSIB, must not disclose protected material to any person’.^134^

This article supports the role of HSSIB in robustly examining systemic risks to patient safety and focusing on learning and improvement rather than assessing blame. Ensuring that HSSIB investigations and reports focus on understanding the causes of safety incidents, rather than appearing to assess civil, criminal, or an individual’s fitness to practise, is entirely appropriate.^135^ However, the proposal to create ‘safe spaces’ which prohibit disclosure of information to anyone, including patients and families, is wrong in principle and problematic in practice.

The principal objection is that it is morally wrong to exclude those most affected by the incident from accessing all available information. As explained in Section III, this article articulates a concept of candour which imposes a private and public duty on clinicians and the health service, respectively. Crucially, the latter includes an obligation to explain *why* patients were harmed. A reform which denies patients and families access to the whole truth is difficult to reconcile with a commitment to candour. Strictly speaking, the proposed ‘safe space’ is not necessarily inconsistent with the duties of candour, in that clause 108 only prohibits HSSIB, and not clinicians or providers of care, from making such disclosures. The new provisions would also not apply to information which is already lawfully in the public domain.^136^ However, it is likely that disclosures which are protected within HSSIB will affect the amount and quality of information provided to patients and families in those affected cases. The proposals clearly contemplate protecting disclosures about safety incidents *beyond* that provided by the statutory duty of candour, and to which patients and their families would be denied access. Ultimately, whilst HSSIB would need to provide patients and families with ‘all relevant information’ relating to their care, ‘all other information’ collected as part of the investigation would be protected from disclosure.^137^

It is also not clear where the line between ‘relevant’ and ‘other’ information will be drawn,^138^ but the basis for making any such distinction is questionable. Patients and families deserve to know not just *what* has happened to them, but *why* it happened. Beyond respecting any confidential personal information in relation to clinicians involved in the delivery of care, it is difficult to understand why other explanatory factors that emerge from a root cause analysis should be withheld from those who have suffered harm. Under these proposals, such explanatory factors, which tend to revolve around resourcing (especially staffing and equipment), decision making, and communication between clinicians, are likely to be deemed as ‘other information’ and so prohibited from being disclosed. Thus, patients and families affected by such incidents will not be able to access the whole truth about what happened to them and why. This will dilute the duty of candour by providing an incomplete explanation of the incident in question. This is wrong in principle as it fails to comply with the public nature of the duty of candour, especially in terms of understanding the underlying reasons which explain harmful events. Whilst HSSIB has a discretion (not a duty) to send draft reports to ‘any other person’ and to take into account their comments,^139^ if that report removes protected material then this is unlikely to satisfy patients or families seeking to understand the whole truth.

The strongest argument in favour of creating safe spaces is that any restriction on truth telling is justified in the public interest. That is, the greater good of learning and ensuring safety improvements potentially benefits everyone who uses the health service, and outweighs the rights of individuals to know the whole truth. Whilst this might seem an acceptable trade-off, it rests on a number of assumptions which lack any underpinning evidence. The first is that the creation of a safe space would necessarily lead to systemic learning and safer healthcare for everyone. However, increased reporting and improved investigations by no means guarantee safer healthcare. Reviews have consistently found little evidence that existing incident reporting systems have improved patient safety or led to desired cultural changes in relation to learning and improvement.^140^ Whilst the safe space proposals are new and attempt to avoid blame-based investigations, as an independent member of the HSIB Advisory Panel has conceded, the notion that this will improve safety is untested with no certainty that such benefits would follow in practice.^141^ Even those who call for independent national incident investigations acknowledge that it remains an early experiment in health policy which faces many practical challenges.^142^

One such challenge is trying to adapt an investigatory model designed for other safety critical industries, such as aviation, to healthcare. Aviation has long had systems for staff to confidentially report safety problems,^143^ and there is evidence that maximising the amount of safety information increases the capacity of organisational learning.^144^ However, the assumption that the success of this model in aviation translates well to healthcare is questionable, given that aviation and healthcare are very different.^145^ The vast majority of aviation incidents reported by staff involve near misses which, by definition, cause no harm and are likely to be much easier for staff to report fully and freely. Healthcare is more diverse and complex than aviation and is also based on intimacy, trust, and compassion, which can render direct comparison with aviation somewhat meaningless.^146^ This is not to deny that healthcare could benefit from the same conditions that have enabled an enhanced safety culture in aviation; for example, sufficient staffing and resources, effective training, teamwork, and the use of checklists.^147^ But the relationship between patients, professionals, and the health service is entirely different to that between airline customers, airline staff, and the aviation service, and the use of ‘protected disclosures’ in aviation investigations does not necessarily mean that these are appropriate for healthcare.

The safe space proposals assume that candour to patients and learning and improvement are somehow mutually exclusive. A better view is that complete candour to patients is the *first step* in terms of the process of learning and improving. That said, there remains a conflict between protecting and supporting staff and thoroughly investigating safety incidents, especially those associated with human error.^148^ There may be good arguments to protect clinicians and healthcare staff from disciplinary proceedings (brought by their employer or professional regulator) where they have provided information about a safety incident, provided there is no evidence of criminality or wilful neglect.^149^ However, it is one thing to declare that such information should ordinarily be inadmissible as evidence for clearly identified disciplinary proceedings, but it is quite another to prohibit disclosure to patients and families directly affected by such harm.^150^ And unless radical reform is taken to abandon the fault-based liability system, patients are rightly able to seek compensation through pursuing clinical negligence proceedings. Indeed, it is noticeable that the Joint Committee report on the original draft Bill somewhat grudgingly accepted that ‘there is nothing unreasonable about injured patients seeking compensation or other redress’,^151^ which raises suspicions about additional cost saving motives for introducing this safe space.

The prohibition on disclosure is not absolute, with a number of exceptions set out in clause 109. The fact that five exceptions are set out in a lengthy schedule made up of eight clauses creates uncertainty and, arguably, undermines the principle of a safe space.^152^ The first two exceptions are relatively uncontroversial and pertain to the ability of HSSIB to effectively discharge its investigatory function; HSSIB can make disclosures necessary for it to carry out this function, both in terms of internal communications (within HSSIB) and also, somewhat vaguely, to those ‘not connected with HSSIB’. Secondly, HSSIB may disclose material necessary for prosecuting or investigating the offences created under the Bill of obstructing or misleading an investigation and of unlawfully disclosing protected material. Strangely, the Bill makes no reference to evidence of specific criminal offences, such as assault, fraud, or manslaughter, as reasons to disclose.^153^

The remaining three exceptions are likely to prove highly problematic in practice. ‘Disclosures relating to safety risks’ allows for disclosures to address a ‘serious and continuing risk to the safety of any patient or to the public’ to those ‘in a position to address the risk’, such as an employer of a healthcare professional.^154^ Given that the intellectual architects of HSSIB envisaged an independent investigator focussing on a small number of serious systemic risks (for example, unsafe levels of staffing, problems with technology and equipment),^155^ it might be expected that HSSIB will routinely handle information that addresses such risks to public safety, and that a faithful interpretation of this clause would require disclosure, albeit not to patients and their families, but to ‘those in a position to address the risk’. Regrettably, an amendment allowing for HSSIB to exercise a discretion to disclose to patients and families (on condition of confidentiality) was defeated.^156^

Disclosure may also be ordered by the High Court if it determines that the interests of justice outweigh any possible adverse impact on encouraging the provision of information and to securing the safety of healthcare services. This risks greater recourse to the legal process by forcing aggrieved patients and families with the financial means to access proceedings, to make such applications. Given that these reforms are seeking to move away from blame and accountability, it is somewhat ironic that this may provoke patients to turn to law. Yet, it is difficult to see how else they can reliably know whether information is being kept from them. Finally, coroners may request disclosure of protected material from HSSIB, consistent with their broad existing powers of obtaining evidence,^157^ and apply to the High Court to disclose this in their reports or to another person in the interests of justice. An attempt by the Parliamentary and Health Service Ombudsman to have similar powers to request disclosure has, thus far, proved unsuccessful. This is despite an opinion that the lack of such powers would violate international standards on the Ombudsman Institution and would be likely to undermine trust in a key institution protecting citizen rights.^158^

Overall, the argument that prohibiting the disclosure of protected material will achieve the stated policy goal of improving safety is open to question on numerous fronts. Given the qualified nature of privilege, with five exceptions set out in clause 109, healthcare professionals are unlikely to feel completely safe in disclosing information. Furthermore, the exceptions are not closed, with the inclusion of a regulation-making power allowing the Secretary of State to create additional exceptions in the future and for ‘a person to exercise a discretion in dealing with any matter’.^159^ Such a broadly drafted provision is unlikely to inspire confidence in encouraging staff to feel safe in disclosing information. The net effect of these proposals is that the safe space is limited and uncertain, and the duty of candour is compromised with those affected denied access by what will look like a ‘secret court’.^160^ Ultimately, it is unlikely that creating such a space will make much of an impact by itself, absent more radical reform to the funding of the health service, safety training and support for staff, and re-orienting the medico-legal system away from a fault-based liability model.^161^

## VI. CONCLUSION

The value of being honest about healthcare harm cannot be overstated. Whilst mistakes and complications are inevitable features of delivering healthcare, there is no justification for preventing patients and their families from understanding the whole truth about harm that has happened to them. Although honesty appears central to the main ethical theories that have been applied to healthcare, formal codes of medical ethics have been strangely silent on the matter. The creation of the professional and statutory duties of candour appropriately signals the importance of truth telling. Whilst the statutory duty is complex, it nevertheless captures the attention of professionals and providers of care in a way that policies and guidance were unable to. It is unduly pessimistic to assume that a compliance focussed approach to encouraging candour will necessarily fail, or that actions against providers for breaching the duty are misguided. This is not to deny that candour confronts deeply rooted cultural commitments to professional identity, reputation, loyalty, and a fear of blame, and it is this which has partly motivated proposals to create so-called safe spaces for investigation, which prohibits disclosure and purportedly protects practitioners.

There is a clear need for an independent safety investigations agency to adopt robust methodologies for examining the full range of factors that contribute to safety failures. In particular, the principles that such investigations are independent and non-punitive are essential to their success. The capacity of agencies to understand and attempt to reduce systemic risks is important, as is their role in discouraging an approach that inappropriately blames individuals. However, the case for making this information legally privileged is wrong in principle and problematic in practice. As a matter of principle, preventing the primary victims of harm from accessing the whole truth is unacceptable. Nobody has a stronger moral claim on this information than those who have been adversely affected by such incidents. In relation to the safe space proposals, it cannot be right for patients to be denied access to new information explaining what happened to them and why. This ultimately undermines the duties of candour, exacerbates harm, and diminishes trust for those seeking to discover the truth. Understanding the reasons for healthcare harm is a crucial part of the public nature of the concept of candour, and those directly affected have a right to know why they suffered harm. Whilst the quality and independence of NHS safety investigations requires improvement, there is no reliable evidence that prohibiting disclosure will improve safety. When comparing healthcare with other ‘safety critical’ industries, such as aviation, it is important to remember how different healthcare is and to be cautious about over-estimating the suitability of applying that model in a caring context.

There are also significant doubts about the extent to which safe spaces will provide the intended level of safety for those with relevant information. The five exceptions envisaged in clause 109 of the Health and Care Bill, alongside a provision for the Secretary of State to create additional exceptions, arguably fatally undermine the safe space from the outset. These provisions are also problematic in how they relate to other parts of the medico-legal system for responding to serious incidents and are unlikely to have the desired effect of making staff feel safe. Such changes are likely to have minimal impact without more fundamental reform of legal and regulatory systems which focus on individual fault. Ultimately, candour should not be contained or compromised and ought to be respected as a cardinal principle governing the conduct of those providing care *and* those investigating harm. Harmed patients and their families deserve to know the whole truth.

## CONFLICT OF INTEREST STATEMENT

None declared.

## Footnotes

1. World Health Organization, ‘Patient Safety: Key Facts’ (13 September 2019) <https://www.who.int/news-room/fact-sheets/detail/patient-safety> accessed 11 January 2022.

2 ibid.

3 L Slawomirski, A Auraaen and N Klazinga, ‘The Economics of Patient Safety: Strengthening a Value-Based Approach to Reducing Patient Harm at National Level’, OECD Health Working Papers No 96 (OECD Publishing 2017).

4 L Rolewicz and B Palmer, ‘The NHS Workforce in Numbers’ (7 September 2021) <https://www.nuffieldtrust.org.uk/resource/the-nhs-workforce-in-numbers> accessed 11 January 2022.

5 K Flott, G Fontana and A Darzi, *The Global State of Patient Safety* (Imperial College 2019) 8.

6 C Yau and others, ‘Clinical Negligence Costs: Taking Action to Safeguard NHS Sustainability’ (2020) 368 British Medical Journal m552.

7 National Audit Office (NAO), ‘Managing the Costs of Clinical Negligence in Trusts’, HC 305 Session 2017–2019, 7 September 2017.

8 For example, W Powell, ‘Robbie’s Law: Lack of Candour—The Impact on Patients and Their Families’ (2014) 20(1–2) Clinical Risk 4–6; J Titcombe, *Joshua’s Story: Uncovering the Morecambe Bay NHS Scandal* (Leeds 2015).

9 T Beauchamp and J Childress, *Principles of Biomedical Ethics* (OUP 2019) 283.

10 ibid. A Campbell, G Gillet and G Jones, *Medical Ethics* (OUP 2001).

11 C Kelly and O Quick, ‘The Legal Duty of Candour in Healthcare: The Lessons of History’ (2019) 70(1) Northern Ireland Legal Quarterly 77.

12 General Medical Council (GMC) and Nursing and Midwifery Council (NMC), ‘Openness and Honesty When Things Go Wrong: The Professional Duty of Candour’ (GMC and NMC 2015).

13 The Health and Social Care Act 2008 (Regulated Activities) 2014, reg 20.

14 For a review of interventions designed to encourage ‘speaking up’ within healthcare, see A Jones and others, ‘Interventions Promoting Employee “Speaking-Up” Within Healthcare Workplaces: A Systematic Narrative Review of the International Literature’ (2021) 125(3) Health Policy 375.

15 <https://bills.parliament.uk/bills/3022> (this is the version of the Bill updated to 19 November 2021) accessed 11 January 2022.

16 V Entwistle and O Quick, ‘Trust in the Context of Patient Safety Problems’ (2006) 20(5) Journal of Health Organization and Management 397.

17
*Montgomery v Lanarkshire Health Board* [2015] UKSC 11.

18 ‘First Do No Harm: The Report of the Independent Medicines and Medical Devices Safety Review’ (2020) <https://www.immdsreview.org.uk/downloads/IMMDSReview_Web.pdf> accessed 11 January 2022.

19 K Syrett, ‘The English National Health Service and the “Transparency Turn” in Regulation of Health Care Rationing’ (2011) 3 Amsterdam Law Forum 101.

20 A Sanchez Graells, ‘Centralisation of Procurement and Supply Chain Management in the English NHS: Some Governance and Compliance Challenges’ (2019) 70 Northern Ireland Legal Quarterly 53.

21 T Minssen, N Rajam and M Bogers, ‘Clinical Trial Data Transparency and GDPR Compliance: Implications for Data Sharing and Open Innovation’ (2020) 47 Science and Public Policy 616.

22 <https://www.gov.uk/government/publications/priority-groups-for-coronavirus-covid-19-vaccination-advice-from-the-jcvi-30-december-2020/joint-committee-on-vaccination-and-immunisation-advice-on-priority-groups-for-covid-19-vaccination-30-december-2020> accessed 11 January 2022.

23 Campbell(n 10) 8, 16; J Oakley, ‘A Virtue Ethics Approach’ in H Kuhse and P Singer (eds), *A Companion to Bioethics* (Blackwell 2001) 86.

24 L Mulcahy, *Disputing Doctors: The Socio-Legal Dynamics of Complaints about Medical Care* (Open University Press 2003) 94.

25 NHS Resolution (NHSR), *Behavioural Insights into Patient Motivation to Make a Claim for Clinical Negligence: Final Report by the Behavioural Insights Team* (NHSR 2018).

26 For example, D Hilfiker, ‘Facing our Mistakes’ (1984) 310 New England Journal of Medicine 118; F Huyler, *The Blood of Strangers: True Stories from the Emergency Room* (Fourth Estate 2001); A Gawande, *Complications: A Surgeon’s Notes on an Imperfect Science* (Profile Books 2003); D Ofri, *What Doctors Feel: How Emotions Affect the Practice of Medicine* (Beacon Press 2013).

27 N Berlinger, ‘“Missing the Mark”: Medical Error, Forgiveness, and Justice’ in VA Sharpe (ed), *Accountability: Patient Safety and Policy Reform* (Georgetown University Press 2004) 119–34, 127. See also N Berlinger, *After Harm: Medical Error and the Ethics of Forgiveness* (John Hopkins University Press 2005).

28 Beauchamp and Childress (n 9).

29 M Barry and S Edgman-Levitan, ‘Shared Decision Making—The Pinnacle of Patient-Centered Care’ (2012) 366(9) The New England Journal of Medicine 780.

30 C Newdick, *Who Should We Treat? Rights, Rationing and Resources in the NHS* (OUP 2005); K Syrett, *Law, Legitimacy and the Rationing of Health Care A Contextual and Comparative Perspective* (CUP 2007).

31 R Higgs, ‘Truth-Telling’ in H Kuhse and P Singer (eds), *A Companion to Bioethics* (Blackwell 2001) 436.

32 E Cave, ‘The Ill-Informed: Consent to Medical Treatment and the Therapeutic Exception’ (2017) 46(2) Common Law World Review 140.

33 For example, if disclosure about medical harm was deemed dangerous to a patient considered at high risk of committing suicide.

34 J Katz, ‘Why Doctors Don’t Disclose Dishonesty’ (1984) 14(1) The Hastings Center Report 35, 35.

35 Department of Health (DH), *An Organisation with a Memory: Report of an Expert Group on Learning from Adverse Events in the NHS* (The Stationery Office 2000); Institute of Medicine, *To Err is Human: Building a Safer Health System* (National Academy Press 2001).

36 M Mello and others, ‘Outcomes in Two Massachusetts Hospital Systems Give Reason for Optimism about Communication-and-Resolution Programs’ (2017) 36(10) Health Affairs 1795; F LeCraw and others, ‘Changes in Liability Claims, Costs, and Resolution Times Following the Introduction of a Communication-and Resolution Program in Tennessee’ (2018) 23(1) Journal of Patient Safety and Risk Management 13.

37 National Patient Safety Agency (NPSA), *Being Open: Communicating Patient Safety Incidents with Patients, Their Families and Carers* (NPSA 2009).

38 NHSR, *Saying Sorry* (NHSR, June 2017).

39 The NHS Constitution for England (1 January 2021) <https://www.gov.uk/government/publications/the-nhs-constitution-for-england/the-nhs-constitution-for-england> accessed 11 January 2022.

40 Y Birks and others, ‘An Exploration of the Implementation of Open Disclosure of Adverse Events in the UK: A Scoping Review and Qualitative Exploration’ (2014) 2(20) Health Services Delivery Research 1, 21.

41 R Harrison and others, ‘Enacting Open Disclosure in the UK National Health Service: A Qualitative Exploration’ (2017) 23(4) Journal of Evaluation in Clinical Practice 713.

42 ibid (n 10).

43 See, eg, W Wade and C Forsyth, *Administrative Law* (11th edn, OUP 2014) ch 12.

44 [1985] 1 WLR 845, 851.

45 [1987] 1 WLR 958, 967.

46
*Montgomery* (n 17).

47 I Kennedy (Chair), *The Report of the Public Inquiry into Children’s Heart Surgery at the Bristol Royal Infirmary 1984–1995: Learning from Bristol* (Cmnd 5207 (I) 2001); DH, *Making Amends: A Consultation Paper Setting out Proposals for Reforming the Approach to Clinical Negligence in the NHS* (DH 2003).

48 reg 20(7) (n 13).

49 See reg 20(5) (n 13) in relation to the statutory duty, and GMC and NMC (n 12) para 12, in relation to the professional duty.

50 Powell (n 8).

51
*Powell v Boladz* (1997) 39 BMLR 35. For a case note on the decision, see Ian Kennedy, *Powell v Boladz* [1998] 6 Medical Law Review 112 (note).

52
*Powell v UK* [2000] ECHR 703.

53 Powell (n 8).

54 GMC, *Good Medical Practice* (GMC 1998) paras 16–18.

55 ibid para 18.

56 GMC, *Good Medical Practice* (GMC 2006) para 30.

57 GMC and NMC (n 12).

58 ibid para 15.

59 ibid para 20.

60
*Report of the Mid Staffordshire NHS Foundation Trust Public Inquiry* (The Stationery Office 2018) recommendation 181.

61 Health (Tobacco, Nicotine, etc and Care) (Scotland) Act 2016 pt 2.

62 Health and Social Care (Quality and Engagement) (Wales) Act 2020 pt 3.

63 Department of Health (Northern Ireland), ‘Implementation Programme for the Recommendations from the Inquiry into Hyponatraemia Related Deaths (IHRD)’ <https://www.health-ni.gov.uk/articles/ihrd-workstream-1-duty-candour> accessed 11 January 2022.

64 Recommended by the Scoping Inquiry into the CervicalCheck Screening Programme, Chaired by Dr Gabriel Scally: <http://scallyreview.ie/> accessed 11 January 2022.

65 D Dalton and N Williams, *Building a Culture of Candour: A Review of the Threshold for the Duty of Candour and of the Incentives for Care Organisations to be Candid* (Royal College of Surgeons 2014).

66 reg 20(7) (n 13).

67 reg 20(2) and (3) (n 13).

68 reg 20(4) (n 13).

69 Dalton and Williams (n 65).

70 reg 20(7) (n 13).

71 reg 20(7) (n 13).

72 CQC, reg 20: *Duty of* *Candour. Information for* *All Providers: NHS Bodies, Adult Social Care, Primary Medical and Dental Care, and Independent Healthcare* (CQC 2015) 9.

73 CQC, ‘The Duty of Candour: Guidance for Providers’ (March 2021) <https://www.cqc.org.uk/guidance-providers/all-services/duty-candour-guidance-providers> accessed 11 January 2022.

74 reg 20(7) (n 13).

75 A Lazare, *On Apology* (OUP 2004) 23.

76 G van Dijck, ‘The Ordered Apology’ (2017) 37(3) Oxford Journal of Legal Studies 562, 566.

77 P Vines, ‘The Value of Apologising within a Moral Community: Making Apologies Work’ (2017) 7(3) Onati Socio-Legal Series 370, 375.

78 Van Dijck (n 76).

79 Lazare (n 75) 1.

80 Vines (n 77) 379.

81 NHSR (n 25) 18.

82 For the idea that apologies may constitute a form of compensation for intangible loss, and also encourage ‘self-help’ for those involved, see R Carroll, ‘Compensation for Intangible Loss: A Closer Look at the Remedial Function of Apologies’, in P Vines and A Akkermans (eds), *Unexpected Consequences of Compensation Law* (Hart 2020) ch 9.

83 Ministry of Justice, Memorandum to the Justice Select Committee, Post-legislative assessment of the Compensation Act 2006, January 2012, para 62.

84 G Leung and G Porter, ‘Safety of Candour: How Protected Are Apologies in Open Disclosure?’ (2019) 365 British Medical Journal l4047.

85 A Fields, M Mello and A Kachalia, ‘Apology Laws and Malpractice Liability: What Have We Learned?’ (2021) 30 British Medical Journal Quality and Safety 64.

86 A Bullock and others, *A Review of Research into Health and Care Professional Regulation* (Professional Standards Authority for Health and Social Care 2020).

87 A Gallagher and R Jago, *A Typology of Dishonesty Illustrations from the PSA Section 29 Database* (Professional Standards Authority for Health and Social Care 2017) 25.

88 Professional Standards Authority, *Can Professional Regulation Do More to Encourage Professionals to be Candid When Healthcare or Social Work Goes Wrong? Advice to the Secretary of State for Health* (Professional Standards Authority for Health and Social Care 2013).

89 Professional Standards Authority, *Telling Patients the Truth When Something Goes Wrong: Evaluating the Progress of Professional Regulators in Embedding Professionals’ Duty To Be Candid to Patients* (PSA 2019).

90 Medical Defence Union, Press Release 4 July 2009.

91 National Advisory Group on the Safety of Patients in England, ‘A Promise to Learn—A Commitment to Act, Improving the Safety of Patients in England’ (DH 2013).

92 D Studdert and M Mello, ‘In From the Cold? Law's Evolving Role in Patient Safety’ (2019) 68(2) DePaul Law Review 421.

93 L Gostin and others, ‘The Legal Determinants of Health: Harnessing the Power of Law for Global Health and Sustainable Development’ (2019) 393(10183) Lancet 1857; H Genn, ‘When Law Is Good for Your Health: Mitigating the Social Determinants of Health through Access to Justice’ (2019) 72(1) Current Legal Problems 159.

94 P Walsh, ‘Challenges and Opportunities for Patient Safety and Justice in the UK’ (2018) 23(1) Journal of Patient Safety and Risk Management 7.

95 Health Education England, ‘Patient Safety Syllabus’ <https://www.hee.nhs.uk/our-work/patient-safety> accessed 11 January 2022.

96 M Mello and K Zeiler, ‘Empirical Health Law Scholarship: The State of the Field’ (2008) 96 Georgetown Law Journal 649; P Case, ‘The Jaded Cliche of “Defensive Medical Practice”: From Magically Convincing to Empirically (Un)convincing?’ (2020) 36(2) Journal of Professional Negligence 49.

97 Care Quality Commission (CQC), *Regulation 20: Duty of* *Candour. Information for all Providers: NHS Bodies, Adult Social Care, Primary Medical and Dental Care, and Independent Healthcare* (CQC 2015) 9. Note that this guidance was updated in March 2021 <https://www.cqc.org.uk/guidance-providers/all-services/duty-candour-guidance-providers> accessed 11 January 2022.

98 H Blythe, *Regulating the Duty of Candour: A Report by Action against Medical Accidents on CQC Inspection Reports and Regulation of the Duty of Candour* (Action against Medical Accidents 2015) 5.

99 D Negri, *Regulating the Duty of Candour: Requires Improvement. A Report by Action against Medical Accidents on CQC Inspection Reports and Regulation of the Duty of Candour* (Action against Medical Accidents 2018) 3.

100 National Advisory Group on the Safety of Patients in England, *A Promise to Learn—A Commitment to Act, Improving the Safety of Patients in England* (DH 2013) 11.

101 Dalton and Williams (n 65) 17.

102 I Ayres and J Braithwaite, *Responsive Regulation: Transcending the Deregulation Debate* (OUP 1992).

103 Negri (n 99) 11–14.

104 K Hawkins, *Law as Last Resort: Prosecution Decision Making in a Regulatory Agency* (OUP 2003).

105 For example, the number of criminal actions brought by the CQC (for all areas) increased from 159 in 2017–18 to 211 in 2018–19: CQC, *Annual Report and Accounts 2018/19* (CQC 2019) 16.

106 CQC, ‘Bradford Teaching Hospitals Fined for Failure to Comply with Duty of Candour’ (17 January 2019) <https://www.cqc.org.uk/news/releases/bradford-teaching-hospitals-fined-failure-comply-duty-candour> accessed 11 January 2022.

107 CQC, ‘Trust Fined for Failures in Complying with Duty of Candour Regulation’ (9 October 2019) <https://www.cqc.org.uk/news/releases/trust-fined-failures-complying-duty-candour-regulation> accessed 11 January 2022.

108 C Dyer, ‘Plymouth Trust Is First to be Fined for Breaching Duty of Candour Rules’ (2020) 370 British Medical Journal m3737.

109 The Duty of Candour Procedure (Scotland) Regulations 2018, reg 8.

110 A Wu, ‘Medical Error: The Second Victim’ (2000) 320 British Medical Journal 726.

111 G Currie and others, ‘Narratives of Professional Regulation and Patient Safety: The Case of Medical Devices in Anaesthetics’ (2009) 11(2) Health, Risk & Society 117.

112 G Martin, S Chew and M Dixon-Woods, ‘Senior Stakeholder Views on Policies to Foster a Culture of Openness in the English National Health Service: A Qualitative Interview Study’ (2019) 112(4) Journal of the Royal Society of Medicine 153, 157.

113 See O Quick, *Regulating Patient Safety: The End of Professional Dominance?* (CUP 2017).

114 NHSR, *Annual Report and Accounts 2020/21* (NHSR 2021) 15.

115 NAO (n 7).

116 A Morris, ‘“Common Sense Common Safety”: The Compensation Culture Perspective’ (2011) 27(2) Journal of Professional Negligence 82.

117 DH, *Improving Patient Safety and Openness—The Role of the NHS Litigation Authority in Incentivising the Duty of Candour* (DH 2015).

118 NHSR, ‘What We Do’ (11 March 2020) <https://resolution.nhs.uk/about/our-work/> accessed 11 January 2022.

119 NHSR (n 114) 46.

120 NHSR, *Five Years of Cerebral Palsy Claims: A Thematic Review of NHS Resolution Data* (NHSR 2017).

121 NHSR, *Maternity Incentive Scheme—Year Three. Revised Safety Actions* (NHSR 2021) 62.

122 NHSR, ‘Early Notification Scheme’ (4 November 2021) <https://resolution.nhs.uk/services/claims-management/clinical-schemes/clinical-negligence-scheme-for-trusts/early-notification-scheme/> accessed 11 January 2022.

123 ibid (n 36).

124 NHSR, *The Early Notification Scheme Progress Report: Collaboration and Improved Experience for Families* (NHSR 2017) 31.

125 NHSR, ‘Update on Revisions to Maternal and Perinatal Reporting Requirements’ (September 2020) <https://resolution.nhs.uk/wp-content/uploads/2020/09/September-update-letter-re-paused-EN-Revised-Reporting-Requirements.pdf> accessed 11 January 2022.

126 See J Legemaate, ‘Blame Free Reporting’ in J Tingle and P Bark (eds), *Patient Safety, Law Policy and Practice* (Routledge 2011) 85–96 for discussion of qualified privilege approaches in Australia, Denmark, and the Netherlands.

127 See, eg, concerns surrounding the case of Dr Hadiza Bawa-Garba discussed in detail in R Ameratunga and others, ‘Criminalisation of Unintentional Error in Healthcare in the UK: A Perspective from New Zealand’ (2019) 364 British Medical Journal l706.

128 S Dekker, *Just Culture: Balancing Safety and Accountability* (Ashgate 2007).

129 Department of Health and Social Care, Integration and Innovation: Working Together to Improve Health and Social Care for All (DHSC February 2021). The proposals were initially presented in the Health Services Investigations Bill 2017, which stalled after its second reading in the House of Lords in 2019.

130 <https://www.hsib.org.uk/> accessed 11 January 2022. Note that HSIB applies in England only.

131 House of Lords House of Commons Joint Committee on the Draft Health Service Safety Investigations Bill, Draft Health Service Safety Investigations Bill: A new capability for investigating patient safety incidents Report of Session 2017–19 HL Paper 180, HC 1064 (2018) 9.

132 C Macrae and C Vincent, ‘Learning from Failure, the Need for Independent Safety Investigations in Healthcare’ (2014) 107(11) Journal of the Royal Society of Medicine 439.

133 DH, *Providing a Safe Space in Healthcare Safety Investigations* (DH 2016) 3.17.

134 Protected material is defined in cl 108(2) as ‘any information, document, equipment or other item’ which is held by HSSIB and relates to the incident (n 15).

135 cls 96(4) and 99(4) (n 15) state that neither investigations nor HSSIB reports should assess or determine blame, civil or criminal liability, or whether regulatory action is needed against an individual. It should be noted that such an organisation could not, in any event, determine such matters, which would fall to other decision makers.

136 cl 108(2)(c) (n 15).

137 House of Lords House of Commons Joint Committee (n 131) 11.

138 The clauses in the Bill are silent on this point.

139 cl 101 (n 15).

140 I Mitchell and others, ‘Patient Safety Incident Reporting: A Qualitative Study of Thoughts and Perceptions of Experts 15 years after “To Err is Human”’ (2016) 25 British Medical Journal Quality & Safety 92; C Stavropoulou, C Doherty and P Tosey, ‘How Effective Are Incident-Reporting Systems for Improving Patient Safety? A Systematic Literature Review: Incident-Reporting Systems for Improving Patients’ Safety’ (2015) 93 Milbank Quarterly 826.

141 Written evidence submitted by Professor Murray Anderson-Wallace: <http://data.parliament.uk/writtenevidence/committeeevidence.svc/evidencedocument/draft-health-service-safety-investigations-bill-committee/draft-health-service-safety-investigations-bill/written/84858.html> accessed 11 January 2022.

142 C Macrae, ‘Investigating for Improvement? Five Strategies to Ensure National Patient Safety Investigations Improve Patient Safety’ (2019) 112(9) Journal of the Royal Society of Medicine 365.

143 For example, the Confidential Human Factors Reporting Programme (CHIRP) which has been operating since 1982, see <https://www.chirp.co.uk/> accessed 11 January 2022.

144 M Tamuz, ‘Learning Disabilities for Regulators: The Perils of Organizational Learning in the Air Transportation Industry’ (2001) 33(3) Administration & Society 276.

145 N Kapur and others, ‘Aviation and Healthcare: A Comparative Review with Implications for Patient Safety’ (2016) 7(1) Journal of the Royal Society of Medicine 1.

146 C Macrae and K Stewart, ‘Can We Import Improvements from Industry to Healthcare?’ (2019) 364 British Medical Journal I1039.

147 See Kapur and others (n 145) and Macrae and Stewart (n 146).

148 C Vincent and L Page, ‘Aftermath of Error for Patients and Health Care Staff’ in B Hurwitz and A Sheikh (eds), *Health Care Errors and Patient Safety* (Wiley-Blackwell 2009) 179–92, 190.

149 HSSIB reports will be inadmissible in proceedings to determine civil and criminal, employment tribunals, and to the investigations of regulators, unless authorised by a High Court judge, cl 103(3).

150 Although maintaining this distinction would admittedly be difficult in practice.

151 House of Lords House of Commons Joint Committee (n 131) 25, para 54.

152 sch 14, Health and Care Bill <https://publications.parliament.uk/pa/bills/cbill/58-02/0183/210183.pdf> accessed 11 January 2022.

153 Note that the original version in cl 29(2) of the 2017 Bill made reference to ‘evidence of the commission of an offence’ <https://assets.publishing.service.gov.uk/government/uploads/system/uploads/attachment_data/file/645961/Draft_bill_health_service_safety_investigations_bill.pdf> accessed 11 January 2022.

154 It remains unclear whether this might also extend to health professional regulators, but they are not mentioned in the Explanatory Notes accompanying the Bill: <https://publications.parliament.uk/pa/bills/cbill/58-02/0140/en/210140en.pdf> accessed 11 January 2022.

155 Macrae and Vincent (n 132).

156 HC Deb 26 October 2021, cols 599–630 <https://hansard.parliament.uk/Commons/2021-10-26/debates/aaf875b0-2fc5-4154-a766-1d543164cdb7/HealthAndCareBill(SixteenthSitting)> accessed 11 January 2022.

157 sch 5, Coroners and Justice Act 2009.

158 Parliamentary and Health Service Ombudsman, ‘Ombudsman Welcomes Venice Commission’s Opinion on Health and Care Bill’ (19 October 2021) <https://www.ombudsman.org.uk/news-and-blog/news/ombudsman-welcomes-venice-commissions-opinion-health-and-care-bill> accessed 11 January 2022.

159 cl 109(4) (n 15).

160 J McHale, ‘Patient Safety, the “Safe Space” and the Duty of Candour: Reconciling the Irreconcilable?’ in J Tingle, C O’Neill and M Shimwell (eds), *Global Patient Safety: Law, Policy and Practice* (Routledge 2018) 77–98, 91.

161 The option of reforming or abandoning the clinical negligence system has been a long-standing source of academic and policy debate, albeit that any radical changes remain unlikely. The House of Commons Health and Social Care Committee is currently exploring the subject of NHS Litigation Reform: <https://committees.parliament.uk/call-for-evidence/590> accessed 11 January 2022.

